# Mycotoxins Exposure in Cabinda, Angola—A Pilot Biomonitoring Survey of Breastmilk

**DOI:** 10.3390/toxins14030204

**Published:** 2022-03-10

**Authors:** Sofia Duarte, Liliana J. G. Silva, André M. P. T. Pereira, Marta Gimbi, Cristiane Cesar, Vanessa Vidal, Rita Basílio, Anabela Almeida, Celeste Lino, Angelina Pena

**Affiliations:** 1 LAQV, REQUIMTE, Laboratório de Bromatologia e Farmacognosia, Faculdade de Farmácia da Universidade de Coimbra, Polo III, Azinhaga de Sta Comba, 3000-548 Coimbra, Portugal; s.cancela.duarte@gmail.com (S.D.); andrepereira@ff.uc.pt (A.M.P.T.P.); kudeiagimbi@gmail.com (M.G.); criscurci@gmail.com (C.C.); vanessadvidal@hotmail.com (V.V.); basilio.rita19@gmail.com (R.B.); clino@ff.uc.pt (C.L.); apena@ci.uc.pt (A.P.); 2 Centro de Investigação Vasco da Gama (CIVG), Escola Universitária Vasco da Gama (EUVG), Campus Universitário, Av. José R. Sousa Fernandes, 3020-210 Coimbra, Portugal; almeida.anabela@gmail.com; 3 CIBIT—Coimbra Institute for Biomedical Imaging and Translational Research, Universidade de Coimbra, 3000-548 Coimbra, Portugal

**Keywords:** aflatoxins, Africa, Angola, breast milk, exposure, ochratoxin A, mycotoxin, risk, zearalenone

## Abstract

Breast milk is considered the ideal form of nutrition for newborns and infants. However, it can carry over contaminants, namely mycotoxins, with biological effects to which this population is particularly vulnerable. Human biomonitoring and surveillance programs are particularly scarce in low-income countries, where food security is a more urgent priority in comparison with food safety. This pilot survey aims to assess exposure of breastfed infants to aflatoxin M1 (AFM1), zearalenone (ZEN), and ochratoxin A (OTA) in Angola, and to evaluate the main socio-demographical and food consumption determinants of lactating mothers. All 37 breast milk samples analyzed are found to be contaminated with ZEN and OTA, although none are found contaminated with AFM1. Contamination levels are lower than previously reported for ZEN but higher in the case of OTA. A significant association between ZEN levels in breast milk and the consumption of cookies by the lactating mothers is found. As for OTA, higher levels are observed in the milk from mothers with younger infants, for which high estimated daily intake (EDI) is determined. As far as the authors are aware, this is the first survey of the occurrence of mycotoxins in breast milk in Angola, so further human biomonitoring works should follow, given that mycotoxins are a global health issue that directly impact the health of populations.

## 1. Introduction

Mycotoxins are toxic secondary metabolites produced by a variety of filamentous fungi that contaminate agricultural products throughout the world [1,2]. They are a large group of naturally occurring toxins, and they are diverse regarding their chemical structure, properties, and adverse biological effects on human health [3]. Three fungal genera are regarded as the most important producers of mycotoxins: *Aspergillus*, *Penicillium*, and *Fusarium* [1,2]. These major food and feed contaminants may occur both pre and postharvest, depending on different factors such as climate conditions and agricultural practices [3]. Although mycotoxins are a global concern, their negative impact on health and the economy is greater in developing countries where poor agricultural practices, warm climates, and improper crop storage conditions are common and thus suitable for fungal growth and toxin formation [4]. In addition, food safety standards or their enforcement are usually weak in these regions, which can lead to more frequent exposure of these populations to higher levels of mycotoxins through their diets [3,5].

The toxicity of mycotoxins to humans and animals through food and feed consumption can cause acute and chronic diseases. Furthermore, contaminated feed does not only impact animal health, but also human health, due to the possible carry-over of mycotoxins to animal-derived products such as milk, meat, and eggs [1].

Aflatoxins (AFs), ochratoxin A (OTA), and zearalenone (ZEN) are amongst the mycotoxins of particular concern to public health due to their high toxicity and prevalence [3]. Aflatoxin B1 (AFB1), produced by the *Aspergillus* species, is the most toxic mycotoxin, inducing genotoxicity, immunotoxicity, and growth impairment. It is considered as one of the most potent hepatocarcinogens [6,7], and thus it is classified as Group 1 by the International Agency for Research on Cancer (IARC) [8]. OTA, produced by the *Penicillium* and *Aspergillus* species, is known as a nephrotoxic agent and has also been shown to be hepatoxic, teratogenic, and immunotoxic in several animal species [9]. The IARC has classified this mycotoxin as Group 2B (possibly carcinogenic to humans) [10]. ZEN, produced by the *Fusarium* species, exhibits a high ability to bind to estrogen receptors thus disrupting the endocrine system [11], so it is classified as Group 3 (not classified as to its carcinogenicity to humans) by the IARC [8]. Once ingested, these mycotoxins are metabolized and, ultimately, can be excreted in milk and urine. Aflatoxin M1 (AFM1), a hydroxylated metabolite of AFB1, is the main AFB1-related compound present in milk, and although it is considered a detoxification byproduct, it exhibits high toxicity and carcinogenicity in humans [6,12]. AFM1, OTA, and ZEN are often used as biomarkers of exposure in several studies to assess infant exposure through breast milk [5].

Breast milk is considered the ideal form of nutrition for newborns and infants due to its unique nutritional composition, immunologic components, and associated health benefits [5]. However, the transfer of mycotoxins from maternal diets to breast milk is of great concern. Infants and young children are particularly vulnerable to the adverse effects of mycotoxins because of their lower detoxification capacity, high metabolic rate, and high food intake per kg of body weight [12]. Nonetheless, it is considered that the health benefits of exclusive breastfeeding likely far surpass the health risks from modest levels of transfer. Particularly in economically less developed regions, breastfeeding is important to consider that the alternatives may lack essential nutrients and other beneficial components, and they can be contaminated with mycotoxins at significantly higher levels than breast milk [3].

Several countries have established regulations to limit human exposure to these contaminants. The European Commission (EC) sets strict maximum levels (ML) for processed cereal-based foods and baby foods as well as dietary foods for special medical purposes intended specifically for infants, which are as follows: AFB1 (0.1 µg/kg), OTA (0.5 µg/kg), and ZEN (20 µg/kg) [13]. Additionally, for infant formulae and follow-on formulae, including infant milk and follow-on milk, the EC defines a ML of 0.025 µg/kg for AFM1 [13]. Other international organizations such as the Codex Alimentarius Commission has not determined MLs for these foodstuffs. Similarly, no maximum limits for mycotoxins are established in Angola.

In Cabinda, the province of Angola where the analyzed samples were collected, the average annual rainfall is 800 mm, and the average annual temperature is 25.0 °C [14]. These are favorable conditions for where some of the most important mycotoxigenic fungi develop and produce mycotoxins. In addition to favorable climatic conditions, low-income countries present a greater risk of contamination due to their lack of conditions for the storage of agricultural crops [6]. Angola is a country undergoing economic recovery after the Civil War period (1975–2002), which resulted in the destruction of the country’s rural infrastructure, social services, and productive capacity [15]. Consequently, it is assumed that the prevailing climate and economic levels in Angola are risk factors for mycotoxin contamination.

Although limits of these natural contaminants in infant foods are very restrictive and controlled by surveilling and controlling foodstuffs, breast milk is comparably rarely evaluated [3]. Given the lack of biomonitoring studies in Angola and considering the potential risk of mycotoxins to breastfed infants, the aim of this study is to assess the exposure of infants to AFM1, OTA, and ZEN through breast milk in Angola and to evaluate the main socio-demographical and food consumption determinants of lactating mothers.

## 2. Results and Discussion

### 2.1. Sociodemographic Data

In total, 37 samples of breast milk were analyzed, and the collections took place in two periods: the first between August and September 2018 (*n* = 18) and the second in August 2019 (*n* = 19). Table 1 shows the main sociodemographic and anthropometric characteristics of the participants.

The breastfeeding mothers included in this study were between 18 and 40 years of age (27 ± 7.08 years). Among these, 27% reported currently caring for their first child, whereas the rest (70%) were multiparous. As for the level of education, more than half of the participants (51%) had completed primary/basic education, followed by participants with secondary education (41%) and only one with a higher education degree (bachelor/graduate). A large part reported being a student (46%), 38% of the mothers were housewives, and only 13% mentioned a profession. As for the breastfed infants, they were between 1 and 18 months of age (6.0 ± 3.5 months), and the average weight at the time of milk sample collection was 7.5 ± 1.8 kg.

### 2.2. Mycotoxin Contamination in Breast Milk

Scientific research with breast milk is of great advantage for evaluating direct ingestion, being relatively easy to collect by a non-invasive method. In comparison, collecting urine from babies, who are still wearing diapers, is extremely difficult. In addition, breast milk can provide an assessment of the exposure of both populations: mothers and children.

The analytical reference methods for the detection of mycotoxins in different types of samples are chromatography-based techniques, which are namely associated with mass spectrometry. However, these are expensive, and they require specialized technicians and sophisticated equipment, which are conditions that are difficult to meet in low-income countries. The ELISA technique is widely used for mycotoxin determination as a screening technique and is currently validated for a variety of biological matrices [1].

#### 2.2.1. Aflatoxin M1

Of the 37 milk samples analyzed, no levels of AFM1 above the LOD (5.0 ng/L) were detected. As far as the authors are aware, this is the first study of the occurrence of AFM1 in breast milk in Angola, so it is not possible to compare it with previous occurrence data in this country. Given the high prevalence of AFM1 in breast milk in African countries reported in recently reviewed previous studies [16,17], it can be considered that the results obtained in the present study are unexpected. Considering similar studies reported previously and summed up in Table 2, it is noticeable that some reported a low level of contamination [18,19,20]. In contrast, other studies [21,22,23,24] have found a high incidence of contamination of breast milk with AFM1.

The differences observed between the various studies, regarding the occurrence and levels of AFM1 contamination, can be attributed to several factors, including environmental factors, food storage conditions, analytical methods used to determine AFM1, socioeconomic factors, and the eating habits of mothers [22,23,24,25]. Regarding the analytical method used, it has been shown that the Limit of Detection (LOD) influences the occurrence of these differences. A higher LOD makes the method less sensitive, which can justify a lower number of positive samples in some studies [5]. Since this was an initial study in Angola, a screening technique (Enzyme-linked immunosorbent assay, ELISA) with an LOD of 5.0 ng/L was applied. For example, compared with the results of Iha et al. [20] (LC; 0.3 ng/L), the levels of AFM1 detected in the only positive samples were considerably lower than the LOD of the present study. Regardless, it is noteworthy that, considering the data obtained in the analysis of the samples from Angola, it was found that the exposure of lactating mothers to aflatoxins was low.

#### 2.2.2. Zearalenone

All of the analyzed samples presented detectable levels of ZEN (>LOD; 60 ng/L), ranging between 72.5 and 1487.4 ng/L, with an average value of 380.7 ± 256.7 ng/L. Similar results were found in a study in Italy [35] and in two studies in Turkey [26,36]. These studies also used the ELISA methodology. Valitutti et al. [37], despite finding higher concentrations than those in the present study, observed a lower incidence. Studies carried out in Iran [27] and Austria [38] did not find positive samples (Table 3).

Regarding the comparison of the occurrence results of the present study with those found in the scientific literature, it appears that studies carried out in Spain [39], Nigeria [30], and Italy [37] found higher values in the studied breast milk samples. However, these authors used different methodologies such as UHPLC-HRMS and LC–MS/MS for the detection and quantification of ZEN. Furthermore, climatic differences and food origins, among others, can explain the large variations between studies. The two studies carried out in Italy [35,37] also show higher mean concentrations than those in our sample. In Spain [39] and Italy [35,37] the maximum concentrations observed are also higher.

The highest mean concentration was observed in primiparous mothers (612 ± 318 ng/L). The highest mean concentration refers to the maternal age group 26–33 years old (436 ± 360 ng/L). The sample with the highest concentration of ZEN (1487.4 ng/L) was also detected in this interval of age. Nevertheless, there is no statistically significant difference between both collection periods (2018 and 2019) regarding ZEN levels. Maternal age, profession, education, and the number of children are not significantly correlated with the content of ZEN in their breast milk. As in the present study, Dinleyici et al. [36] also found no correlation between human milk ZEN levels and maternal age. Massart et al. [35] found statistically significant differences in ZEN concentrations in relation to weight, pre-pregnancy, and postpartum body mass index.

There are no statistically significant differences regarding the association of ZEN concentration in milk with the infant’s age. Concerning the breastfed infants, the milk corresponding to the age group of 7–10 months of age features the maximum ZEN concentration (1487.4 ng/L) as well as the highest average ZEN concentration by age group of the infants (416.2 ± 297.3 ng/L).

Milk from mothers with infants weighing higher than 9.1 present the maximum ZEN concentrations in this study (1487.4 ng/L) as well as the highest mean ZEN concentrations per infant weight group (598 ± 501 ng/L).

Finally, a positive direct correlation (*p* = 0.0003) between biscuit consumption and ZEN concentrations were found. It is also noteworthy that it is usual for mothers to offer cookies to their children after the beginning of food diversification. Previously, Massart et al. [35] did not prove a correlation between maternal diet and concentrations of ZEN in human milk. However, in Turkey the concentrations of ZEN are correlated with the consumption of meat, fish, dried figs, dried apricots, red pepper flakes, and peppers by lactating women [36].

#### 2.2.3. Ochratoxin A

The results show a generalized contamination (100%), with a maximum value of 2527 ng/L. The mean contamination value is 700.1 ± 475.1 ng/L. The occurrence of OTA is higher than previously reported in Nigeria [30], Chile [40], and Brazil [20]. However, it is noteworthy that these studies used analytical techniques other that ELISA for the determination of OTA. As mentioned before, this is one of the various factors that can help explain differences in results reported in different studies. The mean OTA contamination level is one of the highest reported, only surpassed by higher values found in Turkey [26,27] and Iran [41].

Comparing the obtained results with those of previously reported studies, higher values were detected. The average content in a study carried out in São Paulo in Brazil was 20 ng/L [42], in Italy in 2006 it was 30 ng/L [43], and in 2007 it was 10 ng/L [44]. However, the present study presents values lower than those obtained in a study carried out in Iran [27] which obtained an average value of 1090 ng/L of OTA, as shown in Table 4.

Considering that mycotoxin content is related to the mothers’ food consumption, it is important to mention that the lowest value (181.1 ng/L) was determined in milk from a mother who responded that she never or less than once a week consumed nuts, dairy products, bread and cereal flakes, and meat. On the other hand, the highest value (2527.1 ng/L) was collected from a mother who consumed nuts more than seven times a week and who had been a mother for 45 days. Cereal and nuts are frequently contaminated with OTA, as recently reviewed [47].

### 2.3. Exposure and Risk Assessment

Exposure early in life affects health later in life. Infant development can be critically affected by artificial or natural contaminants, including mycotoxins [38]. Infants and children are at risk and are about three times more susceptible than adults to the adverse effects of mycotoxins due to high relationships between intake and body weight and between high metabolic rate and low detoxification capacity [29,40]. Therefore, they may be more sensitive to neurotoxic, endocrine, and immunological effects. In addition, they have more restricted diets [48]. For these reasons, a specific and updated risk analysis for this age group is of great interest [49].

Thus, although breast milk can be a route for infants’ exposure to mycotoxins, the diverse benefits of breastfeeding are thought to outweigh this potential risk [3]. Additionally, the levels of contamination of breast milk may be lower compared to foods introduced after stopping breastfeeding. Although there are market options for nutritionally balanced and controlled infant formulas in the context of food security, in some regions of the world, their access can be limited [3,50]. Djamen et al. [51] reported the quality of breast milk in relation to some infant formulas and complementary foods, claiming the superiority of breast milk regarding mycotoxins. Degen et al. [52] investigated the relationship between maternal intake of mycotoxins—within TDI values—and exposure, via breast milk, in exclusively breastfed children. The investigators made TDI adjustments for the age of infants according to EFSA [51]. The study results demonstrate the safety of exclusive breastfeeding in a continuous exposure scenario respecting TDI.

#### 2.3.1. Aflatoxin M1

Since no levels of AFM1 were detected in the present study (<5.0 ng/L), the EDI to ascertain infant exposure was not determined. However, concerns regarding the risk of exposure to aflatoxins are well founded, particularly regarding children. Since aflatoxins are proven genotoxic and carcinogenic substances, JECFA does not determine a TDI value, stating that even exposure levels below 1 ng/kg body weight/day contribute to the risk of liver cancer [53].

#### 2.3.2. Zearalenone

The 100% incidence of ZEN in breast milk, in the present study, reflects that there was recent ingestion of this mycotoxin by all lactating women. However, we are unable to estimate the maternal daily intake (EDI) because the maternal body weight was not available, and the lactational transfer rate of ZEN is not elucidated in the scientific literature. As presented in Table 5, the EDI of ZEN through breast milk consumption ranges between 10.1 and 159.9 ng/kg bw/day, both corresponding to milk from mothers with infants with a weight equal to or higher than 7 kg. Considering the milk from mothers with infants under 16 weeks of age, higher exposures were determined, namely in the worst-case scenario (280.3 ng/kg bw/day), thus resulting in HI up to 3.4.

Overall, 5.5% of babies are at risk due to possible exposure to ZEN above the adjusted TDI. It should be emphasized that, although ZEN has low acute toxicity, chronic ingestion can result in important effects, as mentioned above. We also cannot disregard the possibility of carrying out restrictive diets, with a low variation of foods eaten by the investigated population.

#### 2.3.3. Ochratoxin A

EDI of OTA for breastfed infants ranges between 27.2 and 248.8 ng/kg bw/day. However, for infants under 16 weeks of age, the EDI values are clearly superior, up to 657.0 ng/kg bw/day.

Given the clear evidence for the carcinogenic effects of OTA, the CONTAM Panel of EFSA [47] recently determined that a health-based guidance value (HBGV) such as TDI should not be established. Thus, the TWI of 120 ng OTA/kg bw as determined previously [47] is no longer valid, so the HI cannot be determined. However, considering the previously reported TWI, the HI is higher than 1 in all case scenarios considered.

The EU Scientific Committee on Food (SCF) and the World Health Organization (WHO) already previously concluded that HBGVs do not apply to populations of infants below the ages of 16 and 12 weeks, respectively. However, considering the infant adjusted TDI of 5.2 ng/kw bw/day [54], the HI of the eight infants up to 16 weeks of age ranges between 8.1 (best-case scenario) and 113.3 (worst-case scenario). The results obtained are of concern, since babies are a vulnerable population group for all the previously mentioned reasons.

## 3. Conclusions

In this pilot biomonitoring survey of breast milk contamination with mycotoxins in Angola, the lactating women were not found to be exposed to AFM1, which lies in contrast with their transversal exposure to both ZEN and OTA, resulting in a transfer to breast milk.

The frequency and levels of contamination of ZEN in the analyzed breast milk samples were lower than reported in most previous studies. A significant association was found between biscuit consumption and the levels of ZEN in breast milk.

OTA occurrence in breast milk was higher than previously reported in terms of frequency and average levels of contamination. Contamination was more evident in the milk from mothers with younger infants and with higher levels associated to the consumption of nuts.

Although the contamination of breast milk does not constitute a reason to discourage the practice of breastfeeding, it does encourage the implementation of measures to prevent food contamination by mycotoxins. Studies that conduct risk assessment are thus needed, particularly in vulnerable populations such as the one enrolled in our study, in order to support policy making aimed at ensuring, maintaining, and improving the safety of humans.

## 4. Materials and Methods

### 4.1. Sampling

Breast milk samples were collected in winter, at two times: the first between August and September 2018, and the second in August 2019. In total, 37 samples were obtained from lactating mothers living in the municipality of Belize in the province of Cabinda, an enclave in the Congo north of the rest of Angolan territory. Samples were obtained at the Health Center, and milk collection was performed by manual extraction, with the help of a nurse, in sterile storage bags. After this collection, the samples were immediately frozen at −20 °C and preserved at a constant temperature and protected from direct light throughout the transport. These conditions were maintained until their analysis in the Laboratory of Cellular and Molecular Biology of the Escola Universitária Vasco da Gama, in Coimbra.

The participants included in this study met the following criteria: breastfeeding without any record of systemic or breast disease, a signed informed consent form, and a completed questionnaire. The defined exclusion criteria were: under 18 years of age and delivery within the last month (to avoid the analysis of colostrum or transitional milk).

The procedures of this work were developed in accordance with the Declaration of Helsinki of the World Medical Association (WMA) [55], the Declaration of Taipei of the WMA on ethical considerations related to health databases and biobanks [56], and also the Oviedo Convention for the Protection of Human Rights and Human Dignity in the Face of Biology and Medicine [57]. This study was approved by the Scientific Council of the Faculty of Pharmacy of the University of Coimbra and by the Ethics and Animal Welfare Committee (CEBEA) of the Vasco da Gama University School (opinion n° 2019/001). The collection of samples was also authorized by the Directorate of the Hospital of Belize, the Municipal Health Department of Belize, and the Provincial Health Department in Cabinda. Participants voluntarily entered this study after signing the informed consent form, in which the objectives and procedures of the study were clarified, and the confidentiality and anonymity of the collected data were guaranteed.

### 4.2. Sociodemographic Data and Food Consumption

For the characterization of the sample, the participants were instructed to fill in a questionnaire upon the collection of breast milk. In the first collection period (in 2018), the applied questionnaire aimed to obtain information on the participant’s age, number of children, educational level, and profession. Data were also obtained on maternal breastfeeding, if exclusive or supplemented with commercial infant formulas, the date of delivery, and the baby’s weight at birth and at the time of milk collection. Additionally, participants completed a semi-quantitative food frequency questionnaire to determine food consumption in the week preceding the collection of the milk sample (seven days prior). The foods included in this questionnaire were: milk, yogurt, coffee, rice, bread, chocolate, cereals, biscuits, cakes, and nuts.

In the second sampling period (in 2019), the applied questionnaire was similar to the previous one, consisting of three parts: (i) individual characteristics, namely age, number of children, date of delivery, and the baby’s weight at the time of milk collection; (ii) sociodemographic data, including education, occupation, and place of residence; and (iii) data on the origin of the food consumed (local products or commercial areas). A semi-quantitative food frequency questionnaire was also filled out, referring to food consumption in the week prior to the collection of the milk sample (seven days before). This questionnaire included the following food groups: dairy products; cereals and derivatives; eggs, meat, and fish; fruit and vegetables; sweets (biscuits and chocolate); and nuts.

### 4.3. Quantification of Mycotoxins

After thawing the samples, they were subjected to centrifugation (3K15 centrifuge, Reagente 5, Porto, Portugal) for 10 min at 3500× *g* and 10 °C. Then, the upper fat layer was completely removed with a Pasteur pipette, and the remaining milk was transferred to a new Eppendorf^®^ tube. It was necessary to repeat this procedure twice, as the samples had a high fat content.

For ZEN determination, given its conjugation with glucuronic acid and sulphate, a previous enzymatic deconjugation step was necessary, so 1 mL of skimmed milk was subjected to enzymatic treatment with 20 μL of β-glucuronidase/Arylsulphatase (from *Helix pomatia*) from Roche Diagnostics GmbH (Germany). It was then vortexed briefly and incubated for 3 h at 37 °C. After the incubation period, 0.1 mL of the sample was removed, and 0.1 mL of methanol was added to 0.9 mL of the hydrolyzed and skimmed sample.

For the determination of mycotoxins in the breast milk samples, the competitive ELISA technique was performed using the commercial kits as follows: AFM1 (RIDASCREEN^®^, R1121, R-Biopharm AG^®^, Darmstadt, Germany), ZEN (RIDASCREEN^®^, R1401, R-Biopharm AG^®^, Darmstadt, Germany), and OTA (RNM 98008, REAGEN^®^, Moorestown, NJ, USA). All the instructions provided by the manufacturers were followed. The standard curve in the enzyme immunoassays, AFM1, ZEN and OTA, was drawn with the mean values of each of the six duplicated concentration levels: 0, 5, 10, 20, 40, and 80 ng/L; 0, 50, 150, 450, 1350, and 4050 ng/L; and 0, 15, 30, 60, 120, 140 ng/L, respectively.

The absorbances obtained were entered into specific software (RIDASOFT^®^ Win.NET version 1.1.1.) from the same manufacturer, and, using the cubic spline function, the AFM1 and ZEN contents in each sample were calculated. For OTA, an EXCEL spreadsheet was provided by the manufacturer.

According to the manufacturers, the detection limit (LOD) corresponded to 5 ng/L (AFM1), 60 ng/L (ZEN), and 150 ng/L (OTA).

### 4.4. Exposure and Risk Assessment

For exposure assessment, a deterministic approach was followed. The estimated daily intake (EDI) of mycotoxins was calculated according to the following formula [54] and expressed in ng/kg body weight/day:

EDI = (Concentration of Mycotoxin × milk consumption)/Body weight(1)

The concentration of mycotoxin corresponds to the content determined in the positive samples (≥LOD), the milk consumption corresponds to the average volume of milk ingested per day by the infants, and the body weight corresponds to the average weight of the infants (expressed in kg). The estimated daily consumption of milk was considered to be 150 mL/kg per day for infants up to 7 kg of body weight and 1 L per day for infants with a weight equal to or higher than 7 kg [12]. For infants up to an age of 16 weeks, because of the highest relative consumption on a body weight basis, the high consumption value of 260 mL/kg bw per day was considered, following EFSA guidance [54].

The mean body weight was calculated considering the infants’ weight at the time of breast milk collection, as reported by the mothers. The weight was not available for one child.

For risk assessment, the hazard quotient (HI—Hazard Index, also known as percentage of tolerable daily intake—%TDI) was calculated according to the following formula:

HI = EDI/TDI(2)

The established TDI for zearalenone was 250 ng/kg bw/day [58]. In the case of infants up to an age of 16 weeks, the established TDI was adjusted with a factor of three to account for the impaired excretory function as well as exclusive breastfeeding (83 ng/kg bw/day) [52,54].

Given the clear evidence for the carcinogenic effects of OTA, the previously established TWI of 120 ng OTA/kg bw [59] is no longer valid, so it was not fully applied except to the worst-case scenario for comparison purposes.

### 4.5. Statistical Analysis

For statistical analysis, GraphPad Prism 6.01 was used. To assess the normality of the data, the D’Agostino–Pearson omnibus, Shapiro–Wilk, and KS tests were applied. Comparisons of two groups of data were made using Student’s *t* test with the Mann–Whitney test. To analyze three or more data sets, ANOVA with Dunn’s multiple comparisons test was used. In correlation tests, the Spearman test was used.

Data were considered statistically significant if *p* < 0.05. The mycotoxin concentrations of the samples were tested to see if there was a correlation with the diet (food groups and weekly frequency: never; 1–2 times a week; 3–4 times a week or 5–6 times a week), parity, age, profession, and maternal education levels. Differences in contamination in the two distinct harvest periods (August 2018 and August/September 2019) and whether infants would be more exposed according to age were also evaluated.

## Figures and Tables

**Table 1:** Main sociodemographic characteristics and anthropometric data of the study population (lactating mothers and infants) and levels (ng/L) of the analyzed mycotoxins.

Variable	Frequency (%)	Mean ± SD	Range (Years)	Average [AFM1] ± SD [Min–Max] (ng/L)	Average [ZEN] ±SD [Min–Max] (ng/L)	Average [OTA] ±SD [Min–Max] (ng/L)
Mother age		26.7 ± 7.1 years	18–40 years	<LOD		
18–25 years	17/37 (45%)				377 ± 219 [72.5–1077.9]	714.5 ± 370.3 [312.9–1791.6]
26–33 years	12/37 (32%)				436 ± 360 [185.2–1487.4]	678.0 ± 363.2 [290.6–1419.4]
≥34 years	7/37 (19%)				310 ± 124 [138.2–476.6]	777.3 ± 832.9 [285.1–2527.1]
No answer	1/37 (3%)				268.3	181.1
Profession		n.a.	n.a.	<LOD		
Student	17/37 (46%)				402.9 ± 300.9 [166.2–1487.4]	591.4 ± 227.7 [313.0–1039.3]
Domestic	14/37 (38%)				368.9 ± 255.2 [72.5–1077.9]	844.2 ± 688.1 [181.1–2527.1]
Teacher	3/37 (8%)				383.0 ± 148.2 [212.2–476.6]	531.5 ± 367.3 [285.1–953.7]
Operator	1/37 (2%)				237.2	1171.1
Tourism technician	1/37 (3%)				317.1	815.5
No answer	1/37 (3%)				369.7	452.1
Education level		n.a.	n.a.	<LOD		
Elementary education	24/37 (65%)				408.0 ± 301.7 [72.5–1487.4]	664.9 ± 392.8 [285.1–1791.6]
High school	10/37 (27%)				357.1 ± 141.5 (212.2–593.7]	642.3 ± 315.5 [313.0–1171.1]
Higher education	1/37 (3%)				317.1	815.5
No answer	2/37 (5%)				203.3 ± 92.0 [138.2–268.3]	1354.1 ± 1658.9 [181.1–2527.1]
**Variable**	**Frequency (%)**	**Mean ± SD**	**Range (Years)**	**Average [AFM1] ±SD** **[Min–Max] (ng/L)**	**Average ^1^ [ZEN] ±SD** **[Min–Max] (ng/L)**	**Average ^1^ [OTA] ±SD** **[Min–Max] (ng/L)**
Number of children		3.0 ± 1.9 children	1–9 children	<LOD		
1	10/37 (27%)				612.0 ± 318.0 [166.2–1077.9]	678.4 ± 215.3 [351.0–1123.2]
2	5/37 (14%)				325.0 ± 148.0 [212.2–575.1]	573.0 ± 293.6 [313.0–953.7]
3	9/37 (24%)				459.8 ± 413.9 [72.5–1487.4]	712.3 ± 485.7 [285.1–1791.6]
≥4	12/37 (35%)				300.2 ± 96.7 [138.2–476.6]	796.2 ± 681.8 [181.1–2527.1]
No answer	1/37 (3%)				600.2	290.6
Infant age		6.0 ± 3.5 months	1–18 months	<LOD		
1–3 months	8/37 (22%)				372.1 ± 305.5 [138.2–1077.9]	800.5 ± 762.1 [181.1–2527.1]
4–6 months	10/37 (27%)				323.1 ± 164.0 [72.5–600.2]	775.2 ± 540.5 [290.6–1791.6]
7–10 months	17/37 (46%)				416.2 ± 297.3 [186.93–1487.4]	579.9 ± 222.7 [297.6–967.4]
≥12 months	2/37 (5%)				401.2 ± 83.5 [342.2–460.2]	945.8 ± 368.8 [685.1–1206.6]
Weight of infants ^2^		7.5 ± 1.8 kg	3.5–11.4 kg	<LOD		
3.5–6.9 kg	10/37 (27%)				299.0 ± 95.0 [138.2–460.2]	886.3 ± 708.0 [181.1–2527.1]
7–9 kg	21/37 (57%)				382.0 ± 216.0 [72.5–1077.9]	679.2 ± 374.3 [290.6–1791.6]
≥9.1 kg	5/37 (14%)				598.0 ± 501.0 [314.9–147.4]	492.7 ± 155.8 [351.0–685.1]
No answer	1/37 (2.7%)				242.52	316.7

^1^ Considering only positive samples (>LOD). ^2^ Corresponds to the weight of infants at the time of collection of breast milk samples. n.a.: not available.

**Table 2:** Occurrence of AFM1 in breast milk samples reported in recent studies.

Country (Year)	Frequency of Contamination (%)	AFM1 Concentration (ng/L)		Analytical Method (LOD ng/L)	Reference
		Mean ± SD	Range		
Angola (2018–2019)	0/37 (0%)	n.a.	<5.0	ELISA (5.0)	Present study
Turkey (2017/2018)	78//79 (98,7%)	3.0 (median)	2.59–3.8	ELISA (2.0)	[26]
Iran (2019)	47/90 (52.2%) 17/90 (18.9%)	6.0 ± 1.5 4.4 ± 1.2	5.16–13.5 6.05–12.1	ELISA (5.0) HPLC (n.a.)	[27]
Nigeria (2019)	225/225 (100%)	4.0 ± 1.1	2.33–7.1	HPLC (n.a.)	[21]
Ecuator (2012–2013)	10/78 (13%)	216 ± 116	53–458	IAC/HPLC-FD (33/23)	[28]
Turkey (2017)	53/100 (53%)	6.4	5.10–8.3	ELISA (5.0)	[29]
Iran (2016)	39/250 (15.6%)	21.0 ± 1.0	11.07–39.3	ELISA (2.3)	[25]
Nigeria	1/75 (1.3%)	<LOQ (87)	n.a.	QuEChERS/LC-MS/MS (43.0)	[30]
Portugal (2015–2016)	22/67 (32.8%)	7.4 ± 1.9	5.1 ± 10.6	ELISA (5.0)	[12]
Lebanon (2015–2016)	104/111 (93.8%)	4.3 ± 1.8	0.22–7.9	ELISA (0.2)	[21]
Iran (2015)	88/88 (100%)	3.2	0.1–13.6	ELISA (0.04)	[23]
Cyprus (2015)	40/50 (80%)	7.8 ± 1.7	5.36–28.4	ELISA (5.0)	[31]
Mexico (2014)	100/112 (89%)	10.4	3.01–34.2	ELISA (0.92)	[32]
Brazil (2013)	5/94 (5.3%)	18 ± 5	13–25	HPLC (4.0)	[19]
Colombia (2013)	45/50 (90%)	5.2	0.9–18.5	HPLC (0.6)	[24]
Brazil (2011–2012)	2/100 (2%)	0.55	0.3–0.8	LC (0.3)	[20]
Jordan (2011)	80/80 (100%)	67.8 ± 4.6	9.7–137.2	ELISA (5.0)	[33]
Egypt (2010)	87/125 (69.6%)	74.4 ± 7.1	7.3–328.6	ELISA (5.0)	[34]

ELISA: enzyme-linked immunosorbent assay; HPLC: High performance liquid chromatography; LC: Liquid chromatography; LOD: limit of detection; MS: mass spectrometry; QuEChERS: Quick, Easy, Cheap, Effective, Rugged and Safe; SD: standard deviation; n.a.: not available.

**Table 3:** Occurrence of ZEN in breast milk samples reported in recent studies.

Country (Year)	Frequency of Contamination (%)	ZEN Concentration (ng/L)		Analytical Method (LOD/LOQ ng/L)	Reference
		Mean ± SD	Range		
Angola (2018/2019)	37/37 (100%)	380.7 ± 256.7	72.5–1487.4	ELISA (60)	Present study
Austria (2015/2016)	0/87 *	n.a.	n.a.	LC-MS/MS (-/32)	[38]
Iran (2019)	0/90 (0%) 0/90 (0%)	n.a. n.a.	n.a. n.a.	ELISA (5) HPLC (5)	[27]
Turkey (2017/2018)	79/79 (100%)	340 (median)	230–510	ELISA	[26]
Nigeria	0/75 (0%)	n.a.	n.a.	QuEChERS/LC-MS/MS (93/190)	[30]
Turkey	90/90 (100%)	173.8 (median)	35.7–682	ELISA	[36]
Italy (2011/2013)	Mothers with celiac disease: 12/275 (4%) Control mothers: 15/178 (8%)	2100 2700	2000–17,000 2000–22,000	IAC/HPLC-FD (-/4000)	[37]
Italy	47/47 (100%)	1130 ± 340	260–1780	ELISA (60) and LC-FD (20/50)	[35]
Spain (2012)	13/35 (37%)	n.a.	2100–14,300	QuEChERS/UHPLC-HRMS	[39]

* Longitudinal assessment of a single newborn on 87 consecutive breast milk samples; ELISA: enzyme-linked immunosorbent assay; FD: fluorescence detection; HPLC: High performance liquid chromatography; HRMS: High-resolution mass spectrometry; IAC: Immunoaffinity chromatography; LC: Liquid chromatography; LOD: limit of detection; MS: mass spectrometry; n.a.: not available; QuEChERS: Quick, Easy, Cheap, Effective, Rugged and Safe; SD: standard deviation; UHPLC: Ultra Performance Liquid Chromatography. n.a.: not available.

**Table 4:** Occurrence of OTA in breast milk samples reported in recent studies.

Country (Year)	Frequency of Contamination (%)	OTA Concentration (ng/L)		Analytical Method (LOD/LOQ ng/L)	Reference
		Mean ± SD	Range		
Angola (2018/2019)	37/37 (100%)	700.1 ± 475.1	181.1–2527.1	ELISA (150)	Present study
Turkey (2017/2018)	-/79	340 (median)	-	ELISA	[26]
Iran (2019)	0/90 (0%) 0/90 (0%)	- -	- -	ELISA (5000) HPLC (5000)	[27]
Portugal (2015–2019)	41/42 (97.6%)	305.5 ± 114	59.4–559.6	ELISA (50)	[45]
Nigeria	11/75 (14.7%)	<LOQ (96)	-	QuEChERS/LC-MS/MS (48)	[30]
Iran (2016–2017)	14/84 (16.6%)	1990 ± 1340	110–7340	ELISA (3000)	[41]
Italy (2011/2013)	Mothers with celiac disease: 6/275 (2%) Control mothers: 1/178 (0.5%)		LOQ-0.123 LOQ-56	IAC/HPLC-FD (-/34)	[37]
Iran (2011)	84/87 (96.6%)	24.57 ± 13.6	1.6–60	ELISA	[46]
Brazil (2011–2012)	66/100 (66%)	4	LOQ-21	IAC/HPLC-FD (0.3/0.8)	[20]
Chile (2008; 2010)				LLE/HPLC-FD (10/30)	[40]
Colostrum (1–6 days)	14/17 (82%)	86 ± 59	LOD-186		
Transition milk (15–30 days)	10/15 (67%)	33 ± 27	LOD-81		
Mature milk (2 months)	6/7 (86%)	27 ± 19	LOD-52		
Mature milk (4 months)	5/6 (83%)	30 ± 14	LOD-43		
Mature milk (6 months)	5/5 (100%)	44 ± 18	18–63		
Iran (2011)	2/136 (2.72%)	115	90–140	HPLC-FD	[44]
Italy (2007)	41/57 (78.8%)	10 ± 15.6	LOD-75.1	IAC/HPLC-FD (0.5/1.0)	[42]

ELISA: enzyme-linked immunosorbent assay; FD: fluorescence detection; HPLC: High performance liquid chromatography; IAC: Immunoaffinity chromatography; LC: Liquid chromatography; LLE: Liquid-liquid extraction; LOD: limit of detection; LOQ: limit of quantification; MS: mass spectrometry; QuEChERS: Quick, Easy, Cheap, Effective, Rugged and Safe; SD: standard deviation.

**Table 5:** Assessment of exposure through estimated daily intake (EDI; ng/kg bw/day) and risk through hazard quotient.

		Breastfed < 7 kg (*n* = 11)		Breastfed ≥ 7 kg (*n* = 25)		Infants < 16 Weeks of Age (*n* = 8)	
		EDI	HI	EDI	HI	EDI ^a^	HI ^b^
Zearalenone	Lowest level (Best-case scenario)	20.7	0.1	10.1	0.1	35.9	0.43
	Average level (Average-case scenario)	44.9	0.2	50	0.6	96.8	1.2
	Highest level (Worst-case scenario)	69	0.8	159.9	1.9	280.3	3.4
Ochratoxin A	Lowest level (Best-case scenario)	27.2	-	37.3	-	47.1	-
	Average level (Average-case scenario)	132.9	-	76.1	-	208.1	-
	Highest level (Worst-case scenario)	379.1	-	248.8	-	657.0	-

^a^ Considering a consumption of 260 mL/kg bW/day (EFSA, 2017); ^b^ Considering the infant adjusted TDI of 83 ng/kg bw/day (EFSA, 2017; Degen et al., 2017).

## Data Availability

The data presented in this study are available in this article.
